# Auditory stimulation modulates amygdala network dynamics

**DOI:** 10.1186/1471-2202-15-S1-P52

**Published:** 2014-07-21

**Authors:** François Windels, Peter Stratton, Pankaj Sah

**Affiliations:** 1 Queensland Brain Institute, The University of Queensland, Brisbane, Queensland, Australia

## 

**Figure 1:**
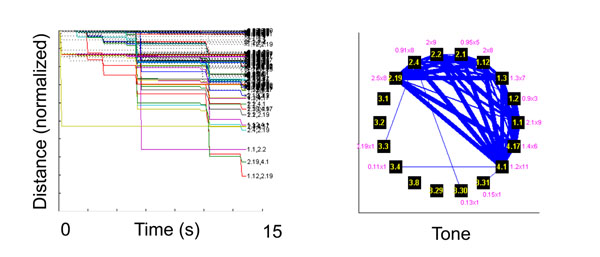
Distance plots (left) show the pairwise correlation (1 ms Gaussian kernel) for every pair of cells recorded on a 1 second basis for the duration of the tone presentation, each colored line shows the dynamic of the interaction between each pair; these results were then used to build the network topology (right) with each unit represented as a node (black square) and the edge strength (blue line thickness) representing the connection strength.

Auditory fear conditioning is a widely used paradigm to study the physiology of associative memory. Based on this model the co-activation of aversive and sensory inputs converging onto neurons of the lateral amygdala is proposed to induce synaptic plasticity that supports fear learning. However, amygdala neurons’ activity entrained by auditory stimulation and the potential ensuing network dynamics remain unexplored. In this study, male Sprague-Dawley rats (n=4) were implanted with 8 tetrodes targeting the lateral amygdala. A screening period started a week after surgery and consisted of repeated presentation of tones at different frequencies (3 to 12 KHz). Z-scored peristimulus-time-histograms were used to analyze the responses of single units to the tones (bins: 20ms; 500ms baseline; p<0.01). We found that half of the tone-responsive cells (n=50) responded to only one frequency whereas the remaining cells responded to up to 8 frequencies. Despite the use of a broad range of frequencies, overlapping the peak sensitivity of the rat audiogram, we did not find a preferential response to one frequency based on the number of responsive cells or the response amplitude. Only 30% of frequency responses, per unit, were observed consistently between recording sessions. We used gravitational clustering [1] to study network dynamics in groups of units recorded concurrently in lateral amygdala (Figure 1). Significant interactions were identified using Monte Carlo simulation. This analysis of small networks showed that response coupling between neurons increased transiently during tone presentation independent of frequency (one-way ANOVA, p<0.01). These results indicate that networks of neurons rather than any single unit represent frequency responses in the amygdala.
